# Microbial Musings – October 2021

**DOI:** 10.1099/mic.0.001129

**Published:** 2021-11-30

**Authors:** Gavin H. Thomas

**Affiliations:** ^1^​ Department of Biology, University of York, York, PO Box 373, UK

## Full-Text

This month we are spoilt for choice for great papers to cover in the *Musings*, and I’m going to start with our latest Microbe Profile on the plant pathogen *Xylella fastidiosa* [1]. These γ-proteobacteria of the family *Xanthomonadaceae* have small genomes of ~2.5 Mb, which is consistent with their survival in limited niches of the plant xylem and the mouthparts of their insect vectors. The article, written by experts Lindsey Burbank (@LindseyBurbank) and Caroline Roper (@RoperLabUCR) from the University of California, Riverside, USA, outlines the most important features of this agricultural pathogen, highlighting the variation of isolates within the species, with some being more like commensals and some devastating pathogens [2]. They describe known aspects of the infection cycle whereby early after infection of xylem cells they use outer-membrane vesicles (OMVs) as decoys to bind sites on the xylem wall to keep the cells in a planktonic state. It is only when they reach high cell densities that adhesion begins and biofilms form, which is an important stage in the colonization of both the plant and the insect mouthparts. The bacterium is also quite unusual in that it can be transmitted by multiple insects and infect multiple hosts, and understanding this is one of the open questions the authors pose at the end of the article about future work that needs to be done on this important endophytic plant pathogen.

For our first research paper this month we look to the group of Ruth Massey (@ProfRuthMassey) from the University of Bristol, UK. I heard Ruth presenting some of her group’s interesting work when she came to York, albeit virtually, to deliver the first James Burgess Memorial Lecture earlier this year. Much of her current work builds on an important large study led by her group that examined the association of *Staphylococcus aureus* genes with bacteraemia (SAB) [3]. In her lecture she emphasized the need to move beyond single virulence factor-based studies based on their findings that clinical outcomes correlate with a broad range of underlying genotypes. In the study they looked across 300 *S *. *aureus* isolates to find positive associations between certain genes and severe disease, using a genome-wide association study (GWAS) approach [3]. In this new study led by Edward Douglas (@ed_douglas13) with Seana Duggan (@SeanaDuggan) and other colleagues in the Massey group, the authors look to determine the function of some of the identified gene products and focus on the MpsB protein [4]. This is a protein that has been studied before in *S. aureus *, primarily by the group of Friedrich Götz in Tübingen, Germany, and it has intriguing functions at the membrane that relate to the generation of membrane potential and bicarbonate/CO_2_ transport as part of the MpsAB complex [5, 6]. In the current paper an *mpsB* mutant strain of *S. aureus* was created that had increased serum survival compared to the wild-type strain, i.e. the presence of the protein sensitizes *S. aureus* to serum. They were able to recapitulate the phenotypes of Gotz’s group of reduced colony size (the small-colony variant, SCV) and also a growth defect at atmospheric CO_2_ levels that could be complemented by 5 % CO_2_. A known phenotype of the SCVs is reduced membrane potential, which they also observed, seeing similar levels to a *hemB* mutant, which reminded me of a paper we published last year by Alastair Hubbard (@dralhubb) and colleagues on a novel *hemA* mutant that also induces SCV formation by a similar mechanism [7]. So why is the presence of MpsB making the cells more sensitive to serum? They propose that this might be due to a role of the membrane potential in resistance to antimicrobial peptides (AMPs) and indeed find that the *mpsB* mutant is more resistant to both the AMPs HNP-1 and LL-37. The mutant was also less cytolytic to human monocytes, which they demonstrate was through the lack of activation of the Agr quorum sensing system, which would explain the reduced production of cytolytic toxins. Finally, they show by titrating levels of the membrane potential, using the uncoupler CCCP, that this can also lead to loss of Agr activation, suggesting that the phenotype of the *mpsB* mutant in relation to toxin production is mediated through the reduced membrane potential that it produces.

Our second paper this month brings us to my home department, with the work of Nathaniel Holman who completed his PhD in York with my colleagues Tony Wilkinson and Maggie Smith (@MaggieCMSmith) and is on the function of the O-linked protein mannosyl-transferase (Pmt) enzyme of *Streptomyces coelicolor *. This is a key enzyme in an unusual system that Maggie discovered a number of years ago when at the University of Aberdeen, UK, when characterizing strains she had isolated that were resistant to infection by bacteriophage ϕC31 [8–10]. The strains also turned out to be resistant to vancomycin, and by trying to understand the relationship between Pmt and antibiotic resistance they were able to identify multiple targets for O-mannosylation in *S. coelicolor *, including some proteins involved in cell wall biosynthesis, the decreased activity of which would explain the increased sensitivity to vancomycin [11]. In this work Nathaniel was trying to understand the structure and function of Pmt, which is an integral membrane protein of unknown structure [12]. He selected 23 residues to be mutated to alanine positions and integrated the mutated genes back onto the chromosome of a *pmt* mutant strain of *S. coelicolor* to assess restoration of Pmt function. From these, the majority had wild-type function, but six failed to complement. While inferences about the roles of these mutated amino acids in enzyme function could be proposed, they correctly checked using Western blotting to see if the non-complementing genes encoded proteins that were detectable and found that they were all undetectable. Returning to these particular amino acids, a series of more or less conservative changes were engineered, but in every case activity was either wild-type when the protein could be seen on a Western blot, or no complementation was seen and the protein was undetected on the Western. There were no intermediate mutants that accumulated in the membrane but had reduced activity. This suggests a very tight coupling of stability of the protein to it having the correct residues at these critical positions – presumably if these important parts of the protein are altered the resulting protein is rapidly degraded, possibly by the FtsH-like protease that is present in *S. coelicolor *. In our group where we routinely do structure/function studies on integral membrane protein transporters and enzymes we have never seen an absolute link like this before, which makes the result unusual in my mind and suggests that there is some particularly strong ‘quality control’ being enacted by the cell here to rapidly remove potentially faulty membrane proteins. As well as this mysterious property of Pmt, the explanation for why *pmt* mutants are resistant to ϕC31 infections remains a mystery, and with Maggie having recently retired from York, the challenge is out there for another budding actinomycete-loving microbiologist to take up!

The last two papers of this month both relate to different aspects of the biology of pathogenic *Escherichia coli *. The first paper is rather special, as it was the first paper to come through our new official rapid review track. Led by Kristian Stærk (@KristianStaerk) from the group of Thomas Anderson from the University of Southern Denmark, Denmark, the paper describes the use of a newly described porcine model of cystitis [13], which they argue is much closer to a human model of urinary tract infection (UTI) than a mouse model of infection. Here they use this model to test the importance of the classical virulence factor for uropathogenic *E. coli* (UPEC), namely the type-1 fimbriae that binds to mannosylated uroplakin receptors on urinal epithelial cells [14]. Using over 30 pigs they infect with either wild-type strain UTI89 or its isogenic *fimH* deletion strain, and they follow urine and blood samples through the 2 week experiments, removing whole bladders at the end of the experiment. While bacteria were able to be recovered from urine in all the pigs, only the wild-type was consistently found infecting the bladder, where in some cases evidence of intracellular bacteria was obtained [15]. While this confirms data seen from mouse models and some more limited studies in humans, it nicely demonstrates the usefulness of this new model, in which they were able to obtain reliable infection with as few as 10^4^ bacteria, reflecting the low inoculum believed to be required in humans. The authors are realistic about the limitations to wide adoption of the model as it requires specialist facilities and staff, but it does add another tool for highly focused work to understand the colonization and persistence of UPEC.

**Fig. 1.**
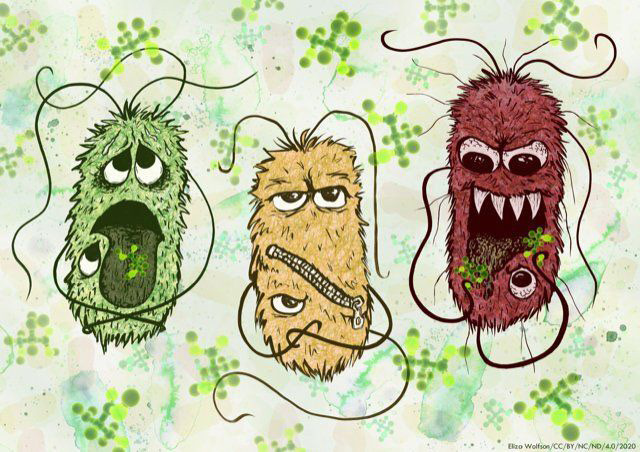
The three faces of *E. coli* pathotypes responding to d-serine, created by Eliza Wolfson. EHEC (left) is stressed by d-serine, UPEC (centre) is fine with it, while NMEC (right), with two copies of the *dsdCXA* catabolic operon, gobbles it up. Source: Eliza Wolfson (CC BY-NC-ND 4.0 2020).

The second related paper also includes UPEC strains in an interesting short communication from James Connolly (@RuaMicro), Jennifer Hallam (@JenniferHallam4), Tom Flett (@tomflett99), Patricia Rimbi (@patricia_rimbi) and colleagues from the group of Andy Roe (@DocAndy_J_Roe), here being led by Nicky O’Boyle (@pogonogo) from the University of Glasgow, UK. This relates to an interesting story from the Roe group in term of the metabolic capabilities of UPEC strains compared to enteroheamorrhagic *E. coli* (EHEC) and neonatal meningitis-associated *E. coli* (NMEC). The three ‘faces’ of these strains are captured by Eliza Wolfson (@eliza_coli), a molecular microbiologist and now full-time scientific illustrator, and I’m sure we’ll see lots of her cool work in multiple places in the future (Fig. 1). The paper builds on Roe’s previous work on the response of *E. coli* to the host-derived molecule d-serine, which both inhibits growth and downregulates the locus of enterocyte effacement (LEE), one of the primary colonization factors of the pathogen [16, 17]. Here they ask the question of whether the same transcriptional responses to d-serine are conserved across the *E. coli* pathotypes and find, rather surprisingly, that there is no single differentially expressed gene common to all three [18]. While EHEC cannot metabolize d-serine, both UPEC and NMEC can, and as such, their growth is not inhibited in the same way, although they still induce many different genes from each other. As they know which genes are responsible for utilization of d-serine, they remove this phenotype in both UPEC and NMEC strains by removing copies of the gene encoding the d-serine-responsive regulator *dsdC* and indeed find that both resulting strains are now sensitive to d-serine and a wide stress-based transcriptional response is now seen by both strains, emphasizing how important the Dsd system is for growth in environments containing d-serine and the large intraspecies differences to it that are found within this important collection of pathogens.

To end this month, I’d like to remember two microbiologists who passed away recently. The first, Professor Pauline Meadow, has a direct and special connection to *Microbiology*, as she was the first female Editor-in-Chief when she led the journal, then the *Journal of General Microbiology* (JGM), from 1981 to 1985. In this period, she was based at University College London, UK, and published in the journal on various topics, including the architecture of the bacterial cell and cell surface using *Pseudomonas* sp. During her leadership the journal made a significant transition in moving to 12 issues a year, which has continued to this day. Born in 1930, she had trained at Oxford as a chemist, one of 8 girls in a class of 100 men, and discovered biochemistry as a supplementary subject which she then studied with Donald Devereux Woods, FRS (1912–1964), one of the founding editors of JGM, and himself a pupil of Marjory Stephenson.

The second microbiologist to remember, and taken from us much more prematurely, is Dr Simon Park, a long-standing member of the Microbiology Society and senior lecturer at the University of Surrey, UK. After an early career working on *Campylobacter* and *Listeria monocytogenes* molecular biology he became more famous for his outreach work, and quite uniquely his interest in microbiology and art, which he blogged about for many years (exploringtheinvisible.com). I became most aware of his work through his captivating 2015 Peter Wildy Prize lecture, which is still a fascinating watch (https://youtu.be/ct2cTsoVUBI) and showcases his diverse activities promoting our subject with many different audiences, including the media, museums and artists with whom he worked over the years. Short tributes to both Pauline Meadow and Simon Park have been published on the Microbiology Society website.
